# Clinical and Pharmacoeconomic evaluation of Fidaxomicin in patients over 65 years of age and immunocompromised patients with recurrent and refractory *Clostridioides difficile* infection

**DOI:** 10.1186/s12879-026-12611-4

**Published:** 2026-01-29

**Authors:** Isabella Alram, Sebastian M. Wingen-Heimann, Marie Engelhard, Nele Pfeiffer, J. Janne Vehreschild, Maria J. G. T. Vehreschild, Sina M. Pütz

**Affiliations:** 1 Department II of Internal Medicine, Infectious Diseases, Goethe University Frankfurt, University Hospital Frankfurt, Frankfurt am Main, Germany; 2 Institute of Translational Research, Cologne Excellence Cluster On Cellular Stress Responses in Aging-Associated Diseases (CECAD), Faculty of Medicine, University Hospital Cologne, University of Cologne, Cologne, Germany; 3 Department I of Internal Medicine, Center for Integrated Oncology Aachen Bonn Cologne Düsseldorf (CIO ABCD) and Excellence Center for Medical Mycology (ECMM), Faculty of Medicine, University Hospital Cologne, University of Cologne, Cologne, Germany; 4 University of Applied Sciences for Economics and Management (FOM), Cologne, Germany; 5 German Centre for Infection Research (DZIF), Partner Site Bonn-Cologne, Cologne, Germany; 6 Department I of Internal Medicine, Faculty of Medicine, University of Cologne, University Hospital Cologne, Cologne, Germany; 7 Faculty of Medicine, Institute for Digital Medicine and Clinical Data Science, Goethe University Frankfurt, Frankfurt am Main, Germany; 8 Division of Infectious Diseases, Department I of Internal Medicine, Faculty of Medicine, University of Cologne, University Hospital Cologne, Cologne, Germany

**Keywords:** Fidaxomicin, *Clostridioides difficile* infection, Disease recurrence, Healthcare resource utilization, Observational study

## Abstract

**Background:**

Recurrent or refractory *Clostridioides difficile* infection (CDI) often affects older and immunocompromised patients, posing clinical and economic challenges. While fidaxomicin has shown lower recurrence rates than vancomycin in the general population, evidence in this population remains limited.

**Methods:**

This retrospective, multicentre case-control study compared patients aged > 65 years or immunocompromised patients receiving fidaxomicin (case group) with those treated with vancomycin (control group) for recurrent or refractory CDI. A microcosting approach was used to assess direct treatment costs.

**Results:**

A total of 344 patients were included (172 per group). Compared to the control group, the fidaxomicin group presented a significantly higher rate of symptom reduction on day 10 (*n* = 163, 95% vs. *n* = 147, 86%; *p* = 0.004) and lower CDI recurrence rates (*n* = 36, 21% vs. *n* = 63, 37%; *p* = 0.001). While the mean CDI treatment costs per patient were significantly higher in the fidaxomicin group (*p* < 0.001), the hospitalisation and overall treatment costs were comparable (€19,898, 95% CI €16,151-€23,645 vs. €20,469, 95% CI €16,837-€24,101, *p* = 0.811; €17,798, 95% CI €14,620-€20,975 vs. €17,300, 95% CI €14,199-€20,400, *p* = 0.840). Key cost drivers were hospitalisation, intensive care unit treatment, and severe initial CDI.

**Conclusions:**

Despite higher drug acquisition costs of fidaxomicin, overall treatment costs were comparable between the two groups with better clinical outcomes in patients treated with fidaxomicin.

**Clinical trial number:**

Not applicable.

**Supplementary Information:**

The online version contains supplementary material available at 10.1186/s12879-026-12611-4.

## Introduction

*Clostridioides difficile* infection (CDI) is a common cause of hospital-acquired infectious diarrhoea in hospitalised patients. Its severity ranges from asymptomatic carriage or diarrhoea to life-threatening pseudomembranous colitis. It is often a complication of broad-spectrum antibiotic treatment [1]. The incidence of and mortality due to CDI is higher in immunocompromised patients compared to the general population – including haematological/oncological patients and patients after solid-organ transplantation [2–5]. This also includes the population of geriatric patients [6]. In these vulnerable patient populations, especially after intensive chemotherapy and under immunosuppressive therapy, a disruption of the gut microbiome is associated with worsened clinical outcomes, including increased infection rates, treatment-related complications, and mortality [7–9].

Fidaxomicin is recommended as first-line treatment for initial CDI in the standard of care setting as well as for patients with a high risk of recurrence [10]. The occurrence of recurrent and refractory CDI is an additional burden for patients, clinicians, and healthcare systems [11, 12]. If a first CDI recurrence occurs, fidaxomicin is recommended if patients are treated with vancomycin during the initial CDI episode [10]. Controversy, however, exists regarding the clinical and health economic impact of fidaxomicin in the context of recurrent and refractory disease [13]. To date, the clinical impact of fidaxomicin has been tested in randomised controlled trials [14–19], whereas the clinical and health economic outcomes have been examined in some single-centre, real-world studies regarding the treatment of mostly initial CDI [20–24]. Studies that specifically investigated patients with recurrent CDI demonstrated that fidaxomicin was associated with significantly lower recurrence rates compared to vancomycin or metronidazole [25, 26]. Although evidence exists regarding the efficacy of fidaxomicin in treating recurrent or refractory CDI, comprehensive real-world data remain limited, especially in immunocompromised patients and patients at advanced age. Moreover, despite its recommendation as first-line therapy in recent guidelines, the use of fidaxomicin remains limited in practice, mainly due to financial constraints which take precedence over clinical prioritisation [27]. We therefore performed a retrospective case-control study at two tertiary-care hospitals in Germany to observe and evaluate clinical and health economic experiences with fidaxomicin for treating recurrent or refractory CDI in immunocompromised patients and patients aged over 65 years compared to vancomycin-treated controls.

## Patients and methods

### Study design and study population

We performed a retrospective, multicentre case-control study at two German university hospitals (Cologne and Frankfurt am Main). We included adult patients with recurrent or refractory CDI initially receiving fidaxomicin (case group) or vancomycin (control group), respectively, between January 2013 and December 2023. Treatment adjustments during the course of therapy were permitted. For inclusion, they needed to present with at least one of the following inclusion criteria: (i) haematological or oncological underlying disease, (ii) diseases requiring immunosuppressive therapy, such as autoimmune disorders or post-organ transplantation status, or (iii) age 65 years or older. The follow-up period was 90 days for in- and outpatients. In the outpatient setting, patients could present for CDI-specific follow-up as well as for management of their underlying comorbidities. Matching of control patients from the same participating study site was performed by using a manually nearest neighbour approach based on the following criteria: age (+/- 15 years), geriatric patients, sex, in- or outpatient status, underlying disease, recurrent vs. refractory CDI, and treatment on an intensive care unit (ICU). Only patients who were alive after at least ten days following treatment initiation with fidaxomicin or vancomycin were included.

### Definitions

Diarrhoea was defined as three or more unformed stools per day. CDI was defined primarily as clinical findings consistent with CDI and microbiological evidence of *C. difficile* toxins by enzyme immunoassay. In addition, cases were identified based on a clinical presentation consistent with CDI in combination with a positive nucleic acid amplification test (NAAT), preferably with a low cycle threshold, or a positive toxigenic *C. difficile* culture, if there was no evidence of another cause of diarrhoea. Furthermore, cases of pseudomembranous colitis confirmed by endoscopy, post colectomy, or autopsy were included, if accompanied by a positive test for the presence of toxigenic *C. difficile*. Recurrence of CDI was defined as a new CDI after initial response to treatment within a period of 90 days, consistent with definitions used in real-world studies and to capture delayed recurrences common among high-risk patient populations [28, 29]. Response to treatment was defined as the absence of diarrhoea for two consecutive days, with improvement continuing throughout the duration of the CDI treatment and no further treatment of CDI required from the second day after completion of the treatment. Refractory CDI was defined as persistence of diarrhoea beyond day 5 of CDI antibiotic treatment or recurrence of diarrhoea on days 5 to 10 of CDI antibiotic treatment after initial cessation of diarrhoea, leading to a therapy change.

### Study objectives and hypotheses

We evaluated the clinical effectiveness of fidaxomicin in treating recurrent and refractory CDI, along with associated healthcare resource utilization and costs. In addition, we examined further outcomes, including time to cure, number of recurrent CDI episodes, and overall length of hospital stay (LOS) in patients treated with fidaxomicin compared with control patients.

### Data documentation

Patient data were collected anonymously into a web-based eCRF, accessible via www.clinicalsurveys.net, after conclusion of the 90-day observation period or after death of the patient. Under the supervision of experienced internal medicine specialists, medical documentation specialists carried out the documentation at the respective study site. The leading study site University Hospital Cologne handled the data monitoring and query management.

### Healthcare resource utilization and cost analysis

The healthcare resource utilization and cost analysis was performed from the perspective of the German healthcare system [30]. It contained direct treatment cost factors that included costs for hospital treatment and costs for CDI medication. Costs for treatment on hospital ward were calculated based on the German Diagnosis Related Groups (G-DRG) systematic provided by Institute for the Hospital Renumeration System für das Entgeltsystem im Krankenhaus (InEK - Institut für das Entgeltsystem im Krankenhaus) and represented personnel and material costs from the years 2013 to 2023 [31]. Costs for CDI medication were calculated by using the pharmacy retail price from 2024 extracted from the Rote Liste^®^, a comprehensive drug directory in Germany [32]. To ensure comparability of hospitalisation and drug acquisition costs, the microcosting approach used year 2024 values expressed in Euros (€). Indirect cost factors, such as productivity losses due to illness-related disability or death before retirement age, were disregarded because of the complex underlying diseases or retirement age. For analyses of hospital length of stay and ward-specific stay durations, only inpatients were considered. As DRG-based cost data were only available for patients treated in inpatient or combined in- and outpatient settings, cost analyses were restricted to these patients, as no standardized cost data were available for outpatients.

### Statistical analysis

The data were processed and analysed using R (R version 4.5.0) [33]. Patient characteristics and outcomes are presented as percentages (absolute numbers) for categorical variables and means (95% confidence interval = 95% CI) for continuous variables. Age and treatment durations are reported as medians (range/inter quartile range). Statistical significance was determined via Pearson’s chi-squared test, Mann-Whitney U Test, Fisher’s exact test or Student’s t-test (two-sided), as appropriate, with *p* < 0.05 as the significance level. Logistic regression and Cox regression analyses were performed via maximum likelihood estimation. A multivariable generalised linear model (GLM) with a gamma distribution and log-link was conducted to assess variables that influence direct treatment costs. Regression analyses included independent patient characteristics and risk factors as well as the underlying CDI treatment. Prior to multivariable analyses, univariate analyses were performed, and all variables with *p* < 0.1 were included in the model. The multivariable analyses reported odds ratios (OR) for logistic regression and GLM or hazard ratios (HR) for Cox regression and 95% CIs; the level of significance was set at *p* < 0.05. Survival analysis was conducted via the Kaplan-Meier method to estimate the cumulative probability of death over time. In accordance with the real-world design of this study, patients with missing values were excluded for the respective analysis step (complete case analysis), if data were missing completely at random.

## Results

### Study population

**Fig. 1:**
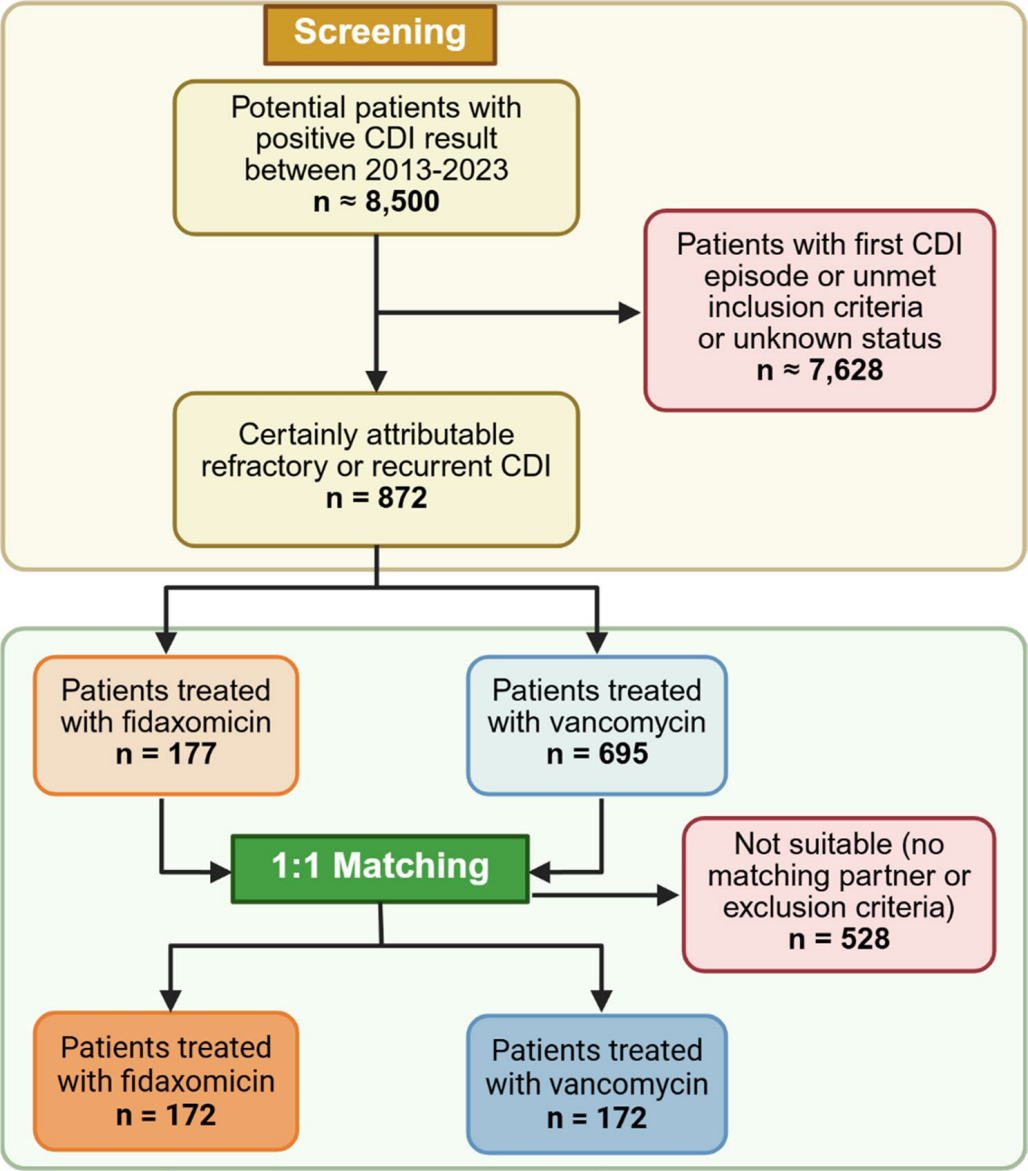
Flow chart of patient selection. Abbreviation: CDI, *Clostridioides difficile* infection. Figure created with biorender

Following the screening of all potential cases within the inclusion period in both centres, a total of 344 patients met the eligibility criteria and were therefore enrolled in the study (Fig. 1). The patient characteristics are demonstrated in Table 1. Approximately one third of the patients presented with refractory CDI and two thirds with recurrent CDI. The most common risk factors were haematological disease, age over 65 years, and a previous hospitalisation within 90 days prior to CDI diagnosis. Almost all patients suffered from mild to moderate CDI, with hospital-acquired infections observed in 42% (*n* = 73) of the fidaxomicin group and 45% (*n* = 78) of the vancomycin group. We found no significant differences between the patient characteristics of the fidaxomicin- and vancomycin-treated patients. For five patients in the fidaxomicin group, it was not possible to find suitable controls, such that matching was carried out across study sites.

**Table 1:** Characteristics of patients with recurrent or refractory *Clostridioides difficile* infection

	Fidaxomicin (*n* = 172)	Vancomycin (*n* = 172)	*p* value
**Female**, n (%)	92 (54)	78 (45)	0.131^a^
**Age**, median (range)	66 (19–85)	67 (19–84)	0.878^b^
**Body mass index**, mean (95% CI)	23.6 (20.0–26.0)	24.6 (21.0-27.8)	0.083^c^
**Caucasian**, n (%)	169 (98)	168 (98)	0.703^a^
**Risk factors**, n (%)			
Haematological disease	62 (36)	56 (33)	0.496^a^
Oncological disease	28 (16)	31 (18)	0.668^a^
Kidney transplantation	24 (14)	22 (13)	0.284^a^
Liver transplantation	6 (4)	3 (2)	0.295^d^
Other underlying disease with immunosuppression	15 (9)	22 (13)	0.330^a^
Geriatric patient > = 65 years	47 (27)	46 (27)	0.903^a^
>= 2 CDI episodes before current CDI diagnosis	19 (11)	15 (9)	0.463^a^
Previous CDI recurrence within 90 days prior to diagnosis	22 (13)	13 (8)	0.109^a^
Previous hospitalisation within 90 days prior to diagnosis	122 (71)	116 (67)	0.484^a^
Severe first CDI episode	16 (9)	11 (6)	0.316^a^
**Other comorbidities**, n (%)			
Cardiovascular disease	114 (66)	116 (67)	0.819^a^
Endocrinological disease	68 (40)	59 (34)	0.315^a^
Gastroenterological disease (except of CDI)	50 (29)	52 (30)	0.813^a^
Nephrological disease	66 (38)	61 (36)	0.576^a^
Psychiatric disease	20 (12)	27 (16)	0.272^a^
Pulmonary disease	39 (23)	27 (16)	0.100^a^
**Type of CDI**, n (%)			
Recurrent CDI	110 (64)	112 (65)	0.822^a^
Refractory CDI	62 (36)	61 (35)	0.910^a^
**Severity of recurrent/refractory CDI**, n (%)			
Mild to moderate	170 (99)	171 (99)	1.000^d^
Severe	1 (1)	1 (1)	1.000^d^
Severe, complicated	1 (1)	0 (0)	1.000^d^
**Number of episodes of recurrent/refractory CDI**,** n (%)**			
1	172 (100)	172 (100)	1.000^a^
2	36 (21)	63 (37)	**0.001** ^a^
3	1 (1)	10 (6)	0.157^d^
4	0 (0)	2 (1)	**0.007** ^d^
**Type of CDI acquisition**, n (%)			0.556^a^
Community-acquired CDI	28 (16)	32 (19)	
Hospital-acquired CDI	73 (42)	78 (45)	
Health system-acquired CDI	59 (34)	50 (29)	
Unknown	12 (7)	12 (7)	

Abbreviations: CI, confidence interval; CDI, *Clostridioides difficile* infection

^a^ Pearson’s chi-squared test, ^b^ Mann-Whitney-U test, ^c^ Student’s t-test, ^d^ Fisher’s exact test

### Treatment types and durations

In the fidaxomicin group, 63% of the patients (*n* = 108) were treated as inpatients, 15% (*n* = 26) as outpatients and 22% (*n* = 38) in an in- and outpatient setting. In the vancomycin group, 58% (*n* = 100) of the patients were inpatients, 14% (*n* = 24) were outpatients, and 28% (*n* = 48) were in- and outpatients. In the inpatient setting of fidaxomicin-treated patients, 96% (*n* = 140) received fidaxomicin standard dose (200 mg twice a day), whereas 2% (*n* = 3) were treated with fidaxomicin extended-pulsed therapy. In 1% (*n* = 1) of the patients, bezlotoxumab or faecal microbiota transfer (FMT) was added to the fidaxomicin treatment, respectively. Outpatients were treated with fidaxomicin standard dose (77%, *n* = 20) or fidaxomicin extended-pulsed therapy (12%, *n* = 3), while bezlotoxumab was added in 15% (*n* = 4) and FMT in 4% (*n* = 1) of patients. Therapy changes and subsequent CDI treatments due to new recurrent or refractory infections after the index episode were documented as well. In the fidaxomicin group, 2% of patients (*n* = 4) received vancomycin following initial therapy with fidaxomicin. Conversely, in the vancomycin group, 7% of patients (*n* = 11) were treated with fidaxomicin after first-line therapy with vancomycin. There were no statistically significant differences in the duration of treatment between the groups (Table 2). Across all recurrent and refractory CDI episodes, fidaxomicin was given orally for a median duration of 11 days (fidaxomicin group: IQR 11–15; vancomycin group: IQR 10–13) in both groups (*p* = 0.367), whereas vancomycin was given orally for a median duration of 13 days (IQR 10–20) in the fidaxomicin group and 12 days (IQR 11–21) in the vancomycin group (*p* = 0.876).

**Table 2:** Treatment details of patients with recurrent or refractory *Clostridioides difficile* infection

	Fidaxomicin group (*n* = 172)	Vancomycin group (*n* = 172)	*p* value
Overall treatment of recurrent/refractory CDI*^,^**,** n (%)**	172 (100)	169 (98)	-
** Fidaxomicin**,** n (%)**	165 (96)	10 (6)	**< 0.001** ^**a**^
Duration (days), Median (IQR)	11 (11–15)	11 (10–13)	0.367^b^
** Fidaxomicin extended pulsed therapy**,** n (%)**	7 (4)	1 (1)	0.067^c^
Duration (days), Median (IQR)	26 (26–27)	13 (na)	0.163^b^
** Vancomycin**,** n (%)**	4 (2)	165 (96)	**< 0.001** ^**c**^
Duration (days), Median (IQR)	13 (10–20)	12 (11–21)	0.876^b^
** Vancomycin tapered pulsed therapy**,** n (%)**	-	12 (7)	-
Duration (days), Median (IQR)	-	49 (36–56)	-
** Metronidazole**,** n (%)**	-	8 (5)	-
Duration (days), Median (IQR)	-	11 (10–14)	-
** Bezlotoxumab**,** n (%)**	5 (3)	-	-
Duration (days), Median (IQR)	26 (11–26)	-	-
**Treatment of first recurrent/refractory CDI**,** n (%)**	172 (100)	172 (100)	-
** Fidaxomicin**,** n (%)**	165 (96)	-	-
Duration (days), Median (IQR)	11 (11–12)	-	-
** Fidaxomicin extended pulsed therapy**,** n (%)**	7 (4)	-	-
Duration (days), Median (IQR)	26 (26–27)	-	-
** Vancomycin**,** n (%)**	-	165 (96)	-
Duration (days), Median (IQR)	-	11 (11–16)	-
** Vancomycin tapered pulsed therapy**,** n (%)**	-	9 (5)	-
Duration (days), Median (IQR)	-	43 (37–51)	-
** Metronidazole**,** n (%)**	-	1 (1)	-
Duration (days), Median (IQR)	-	19 (na)	-
** Bezlotoxumab**,** n (%)**	5 (3)	-	-
Duration (days), Median (IQR)	26 (11–26)	-	-
** FMT**,** n (%)**	2 (1)	-	-
Treatment of subsequent recurrent/refractory CDI*,** n (%)**	172 (100)	172 (100)	-
** Fidaxomicin**,** n (%)**	27 (16)	10 (6)	**0.003** ^**a**^
Duration (days), Median (IQR)	12 (11–14)	11 (10–13)	0.519^b^
** Fidaxomicin extended pulsed therapy**,** n (%)**	-	1 (1)	-
Duration (days), Median (IQR)	-	13 (na)	-
** Vancomycin**,** n (%)**	4 (2)	20 (12)	**0.001** ^**c**^
Duration (days), Median (IQR)	13 (10–20)	11 (10–15)	0.725^b^
** Vancomycin tapered pulsed therapy**,** n (%)**	-	4 (2)	-
Duration (days), Median (IQR)	-	43 (30–54)	-
** Metronidazole**,** n (%)**	-	7 (4)	-
Duration (days), Median (IQR)	-	10 (10–12)	-
** FMT**,** n (%)**	6 (3)	-	-
**Additional antibiotic treatment**			
during CDI treatment, n (%)	95 (55)	105 (61)	0.274^a^
after CDI treatment, n (%)	95 (55)	93 (54)	0.829^a^
**Hospital visits**			
** Inpatient treatment**,** n (%)**	146 (85)	148 (86)	0.760^a^
Number of hospitalisations, Median (Range)	1 (1–5)	1 (1–6)	0.689^b^
Length of initial hospital stay, Median (IQR)	31 (13–55)	33 (14–52)	0.845^b^
Overall hospital length of stay, Median (IQR)	36 (19–55)	37 (19–59)	0.956^b^
** Outpatient treatment**,** n (%)**	64 (37)	72 (42)	0.378^a^
Number of visits, Median (Range)	1 (1–3)	1 (1–3)	**0.025** ^b^

Abbreviations: CI, confidence interval; IQR, interquartile range; LOS, length of stay; CDI, *Clostridioides difficile* infection; na, not applicable

^a^ Pearson’s chi-squared test, ^b^ Mann-Whitney-U test, ^c^ Fisher’s exact test

*within the 90-day observational period, **including the first and the subsequent recurrent/refractory *Clostridioides difficile* infection

With a median of 31 days (IQR 13–55) and 33 days (IQR 14–52) the hospital length of stay of the initial hospitalisation was comparable in the fidaxomicin and vancomycin group (*p* = 0.845) with a similar amount of CDI treatment as the reason for hospitalisation (44%, *n* = 64 vs. 45%, *n* = 67; *p* = 0.845). No significant differences were detected between both groups regarding the number of patients treated in the intermediate care unit (24%, *n* = 35 vs. 24%, *n* = 36; *p* = 0.894) or the ICU (23%, *n* = 34 vs. 22%, *n* = 33; *p* = 0.892) during the observation period as treatment on ICU was part of the matching approach. Patients in both groups had a median of one outpatient visit, with significantly more outpatient visits in the vancomycin group than in the fidaxomicin group (*p* = 0.025).

### Overall effectiveness of fidaxomicin

Symptom reduction on day 10 was reached in significantly more fidaxomicin-treated patients (95%, *n* = 163) than in vancomycin-treated patients (86%, *n* = 147, *p* = 0.004). Univariate logistic regression analysis revealed that fidaxomicin as CDI therapy was the only independent variable with an impact on the symptom reduction on day 10 (OR 3.080, *p* = 0.005; Table 3). The median number of days with CDI-associated diarrhoea was similar in both groups (fidaxomicin group: 5 days, IQR 3–8 days; vancomycin group: 4 days, IQR 3–9 days; *p* = 0.817). A new CDI recurrence within 90 days occurred in significantly more vancomycin-treated patients (37%, *n* = 63) than in fidaxomicin-treated patients (21%, *n* = 36; *p* = 0.001). Overall, there were more recurrent than refractory CDIs (Table 1). The multivariable logistic regression analysis demonstrated that CDI therapy with fidaxomicin significantly reduced the risk of CDI recurrence within the 90-day observational period (OR 0.443, *p* = 0.001), while additional antibiotic treatment after CDI therapy was significantly associated with an increased risk of CDI recurrence (OR 2.221, *p* = 0.002; Table 4).

**Table 3:** Univariate logistic regression analyses of independent variables with impact on *Clostridioides difficile* infection-associated symptom reduction on day 10 based on risk factors for *Clostridioides difficile* infection and *Clostridioides difficile* infection therapy

Variable	*p* value (univariate)	Odds ratio	95% CI
Age	0.493	1.008	0.985–1.029
Fidaxomicin as CDI therapy	**0.005**	3.080	1.440–7.176
Previous CDI recurrence^a^	0.136	0.482	0.194–1.371
Previous hospitalisation^a^	0.326	1.444	0.678–2.975
Severe first CDI episode	0.655	1.404	0.392–8.973
Inpatient treatment	0.630	0.765	0.220–2.054
Additional antibiotic treatment during CDI treatment	0.415	0.736	0.341–1.516

Abbreviations: CDI, *Clostridioides difficile* infection; CI, confidence interval

^a^ within 90 days prior to diagnosis

**Table 4:** Uni- and multivariable logistic regression analyses of independent variables with impact on the occurrence of a new *Clostridioides difficile* infection recurrence within the 90-day observational period based on risk factors for *Clostridioides difficile* infection and *Clostridioides difficile* infection therapy

Variable	*p* value (univariate)	Odds ratio	95% CI	*p* value (multivariate)	Odds ratio	95% CI
Age	0.687	1.003	0.988–1.019	-	-	-
Fidaxomicin as CDI therapy	**0.001**	0.458	0.281–0.737	**0.001**	0.443	0.270–0.719
Previous CDI recurrence^a^	0.252	1.532	0.722–3.141	-	-	-
Previous hospitalisation^a^	0.520	0.849	0.517–1.408	-	-	-
Severe first CDI episode	0.587	1.261	0.523–2.846	-	-	-
Inpatient treatment	0.587	0.836	0.444–1.629	-	-	-
Additional antibiotic treatment during CDI treatment	0.284	1.299	0.808–2.110	-	-	-
Additional antibiotic treatment after CDI treatment	**0.002**	2.146	1.323–3.534	**0.002**	2.221	1.360–3.691

Abbreviations: CDI, *Clostridioides difficile* infection; CI, confidence interval

^a^ within 90 days prior to diagnosis

**Fig. 2:**
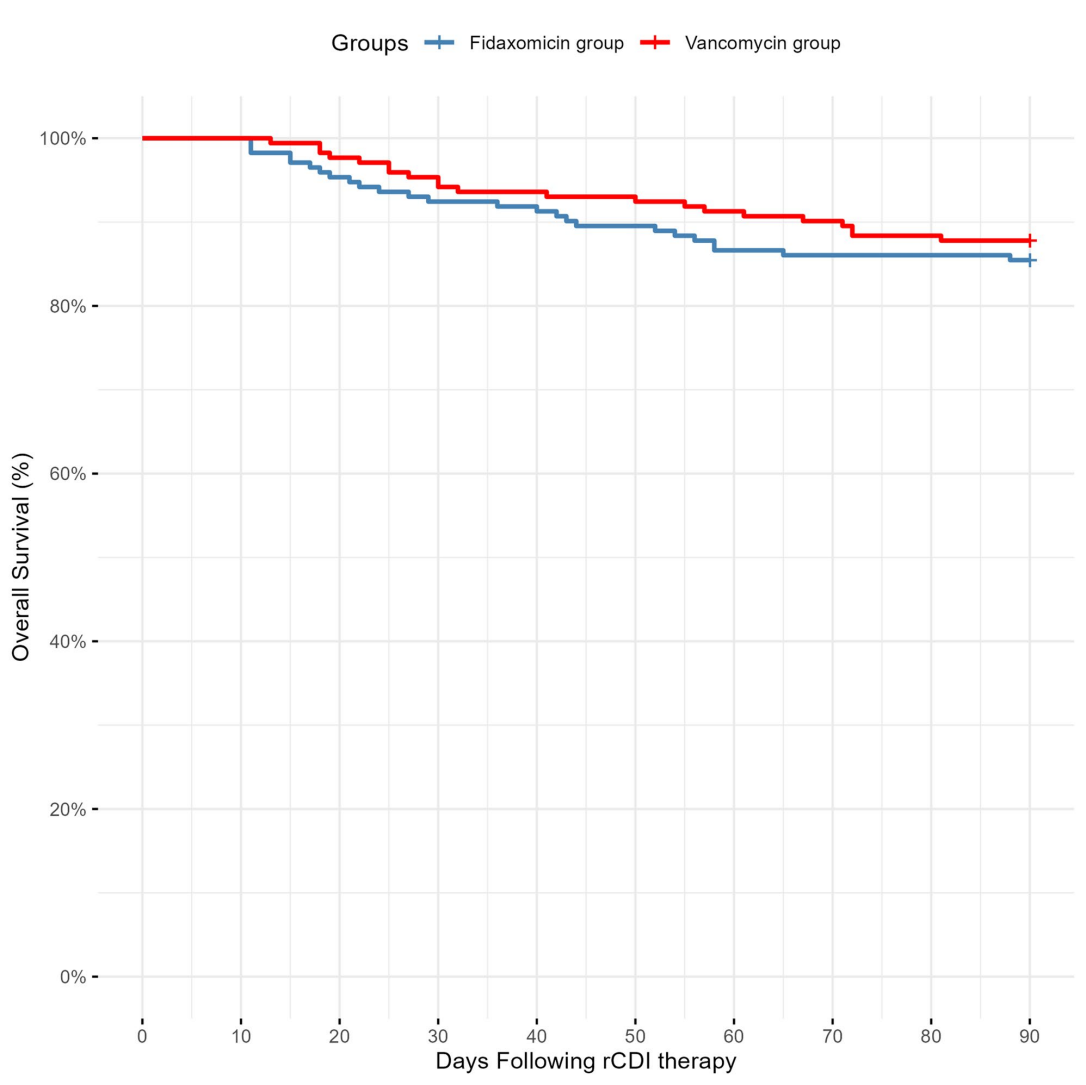
Overall survival of the observed groups. Kaplan-Meier plot shows overall survival rate (in %) until day 90 after therapy initiation with fidaxomicin (blue line) and vancomycin (red line, HR = 0.816, 95% CI: 0.457–1.458, log-rank test: *p* = 0.483). Abbreviations: rCDI, recurrent or refractory *Clostridioides difficile* infection

Overall, 13% of all patients (*n* = 46) died within the 90-day observational period, with no significant difference between the groups (fidaxomicin group: 15%, *n* = 25; vancomycin group: 12%, *n* = 21; *p* = 0.483; Fig. 2). In one fidaxomicin-treated patient, death was attributable to CDI complications and in another patient, death was partially attributable to CDI whereas in the vancomycin group no deaths were attributable to CDI. The death from CDI in the two fidaxomicin-treated patients was associated with another episode of CDI after the successful treatment of the first recurrence with fidaxomicin.

In the subgroup of patients with recurrent CDI, we found similar clinical outcomes (Table S1). In contrast, in the subgroup of patients with refractory CDI, no significant differences in symptom reduction on day 10 or in the rate of recurrence were detected (Table S3). A subgroup analysis excluding the patients who received FMT or bezlotoxumab as first-line therapy for recurrent or refractory CDI yielded results that were consistent with those observed in the overall study population (Table S5).

### Costs for hospitalisation and CDI treatment

Direct treatment costs for recurrent and refractory CDI patients, including hospitalisation and drug acquisition costs, are described in Table 5. For the 294 patients treated in the inpatient and combined in- and outpatient setting, DRG data were available for 91% (*n* = 268) and were therefore used to calculate hospitalisation costs. Mean overall hospitalisation costs were comparable between both groups with €19,898 per patient (95% CI 16,151 − 23,645) in the fidaxomicin group and €20,469 per patient (95% CI 16,837 − 24,101) in the control group (*p* = 0.811). Over 85% of those expenses could be assigned to the initial hospitalisation in both groups, amounting to €17,300 per patient (95% CI 13,596 − 21,005) in the fidaxomicin group and €17,520 per patient (95% CI 13,958 − 21,082) in the control group (*p* = 0.969). Expenses for patients that were hospitalised due to recurrent or refractory CDI were an average of €4,000 higher in the fidaxomicin group compared with the vancomycin group (€8,552 per patient, 95% CI 5,717 − 11,386; €12,578 per patient, 95% CI 9,066 − 16,089, *p* = 0.071).

**Table 5:** Direct treatment costs for patients with recurrent or refractory *Clostridioides difficile* infection in Euro during the 90-day follow-up period

	Fidaxomicin group (*n* = 172)	Vancomycin group (*n* = 172)	*p* value
**Hospitalisation costs**^**I, b**^, **n (%)**	133 (77)	135 (78)	0.795^d^
Costs per patient, mean (95% CI)	19,406 (15,215 − 23,597)	19,300 (15,534 − 23,067)	0.990^a^
**Hospitalisation costs**^**I**^, **n (%) (Year 2024)**	133 (77)	135 (78)	0.795^d^
Costs per patient, mean (95% CI)	19,898 (16,151 − 23,645)	20,469 (16,837 − 24,101)	0.811^a^
**Drug acquisition costs for first recurrent or refractory CDI** ^**c**^			
**Fidaxomicin**,** n (%)**	165 (96)	-	-
Costs per patient, mean (95% CI)	2,020 (1,952 − 2,091)	-	-
**Fidaxomicin extended pulsed therapy**,** n (%)**	7 (4)	-	-
Costs per patient, mean (95% CI)	1,706 (1,647-1,761)	-	-
**Vancomycin**,** n (%)**	-	165 (96)	-
Costs per patient, mean (95% CI)	-	976 (837-1,130)	-
**Vancomycin tapered pulsed therapy**,** n (%)**	-	9 (5)	-
Costs per patient, mean (95% CI)	-	1,272 (730-2,063)	-
**Metronidazole**,** n (%)**	-	1 (1)	-
Costs per patient, mean (95% CI)	-	77	-
**Bezlotoxumab**,** n (%)**	3 (2)	-	-
Costs per patient, mean (95% CI)	2,228 (1,800-2,479)	-	-
**Drug acquisition costs for second recurrent or refractory CDI** ^**c**^			
**Fidaxomicin**,** n (%)**	27 (16)	19 (11)	**0.002** ^**d**^
Costs per patient, mean (95% CI)	2,147 (1,835-2,561)	2,059 (1,806-2,364)	0.699^a^
**Fidaxomicin extended pulsed therapy**,** n (%)**	-	1 (1)	-
Costs per patient, mean (95% CI)	-	1,147	-
**Vancomycin**,** n (%)**	4 (2)	18 (10)	**0.002** ^**d**^
Costs per patient, mean (95% CI)	1,220 (423-2,766)	510 (401–641)	0.161^a^
**Vancomycin tapered pulsed therapy**,** n (%)**	-	2 (1)	-
Costs per patient, mean (95% CI)	-	582 (384–779)	-
**Metronidazole**,** n (%)**	-	6 (3)	-
Costs per patient, mean (95% CI)	-	42 (37–47)	-
**Drug acquisition costs for third recurrent or refractory CDI** ^**c**^			
**Vancomycin**,** n (%)**	-	2 (1)	-
Costs per patient, mean (95% CI)	-	2,919 (538-5,301)	-
**Vancomycin tapered pulsed therapy**,** n (%)**	-	2 (1)	-
Costs per patient, mean (95% CI)	-	850 (818–882)	-
**Metronidazole**,** n (%)**	-	1 (1)	-
Costs per patient, mean (95% CI)	-	24	-
**Overall recurrent/ refractory CDI drug acquisition costs**^**c**^, **n (%)**	172 (100)	169 (98)	0.248^e^
Costs per patient, mean (95% CI)	2,412 (2,254-2,594)	1,256 (1,074 − 1,453)	**< 0.001** ^a^
**Overall direct treatment costs**^**b, c**^, **n (%)**	172 (100)	172 (100)	-
Costs per patient, mean (95% CI)	17,417 (13,935 − 20,899)	16,382 (13,220 − 19,545)	0.676^a^
**Overall direct treatment costs**,** n (%) (Year 2024)**	172 (100)	172 (100)	-
Costs per patient, mean (95% CI)	17,798 (14,620 − 20,975)	17,300 (14,199 − 20,400)	0.840^a^

Abbreviations: CI, Confidence interval; CDI, *Clostridioides difficile* infection

^I^ n-values differ due to missing data; ^a^ Bootstrapped t-test (independent samples, two sided); ^b^ Based on G-DRGs from 2013 to 2023; ^c^ Based on Rote Liste^®^ prices from 2024; ^d^ Pearson chi-square test (two-tailed); ^e^ Fisher´s exact test

Costs for CDI treatment with fidaxomicin and vancomycin were calculated stratified by CDI episode. For initial recurrent or refractory CDI treatment, a mean of €2,020 (95% CI 1,952 − 20,091) and €1,706 (95% CI 1,647-1,761) per patient was spent on fidaxomicin and fidaxomicin extended pulsed therapy. Overall, CDI drug acquisition costs were significantly higher in the fidaxomicin group, with a mean cost of €2,412 per patient (95% CI 2,254-2,594), compared with the vancomycin group, with a mean cost of €1,256 per patient (95% CI 1,074 − 1,453, *p* < 0.001). With regard to the overall direct treatment costs, expenses were similar in both groups (fidaxomicin group: €17,417 per patient, 95% CI 13,935 − 20,899; vancomycin group: €17,300, 95% CI 14,199 − 20,400; *p* = 0.840).

Expenses of €2,180 (95% CI 1,933-2,486) and €1,928 (95% CI 1,487-2,405) per patient in the fidaxomicin and vancomycin groups were associated with drug acquisition costs in the outpatient setting (*p* = 0.367). For inpatient treatment and in- and outpatient treatment, significantly higher mean costs per patient were spent for CDI drug acquisition in the fidaxomicin group (€2,453, 95% CI 2,270-2,656) compared with the vancomycin group (€1,145, 95% CI 956-1,354; *p* < 0.001). When the direct cost parameters were separated for recurrent or refractory CDI patients, the results were comparable to those of the total study cohort (Table S2, Table S4).

Variables influencing overall direct treatment costs of recurrent or refractory CDI patients are given in Table 6. The multivariable GLM revealed that inpatient treatment (OR 4.944, *p* < 0.001), ICU treatment (OR 2.119, *p* < 0.001), hospital-acquired CDI (OR 1.802, *p* < 0.001), rehospitalisations (OR 1.576, *p* < 0.001) and a severe first CDI episode within 90 days prior to recurrent or refractory CDI diagnosis (OR 1.452, *p* = 0.038), were associated with higher overall direct treatment costs. Older age, CDI therapy, type of CDI, subsequent CDI episodes and symptom reduction on day 10 were not found to influence overall costs.

**Table 6:** Generalised linear model of variables influencing overall treatment costs in patients with recurrent or refractory *Clostridioides difficile* infection

Overall treatment costs	Univariate GLM			Multivariable GLM		
	*p*	OR	95% CI	*p*	OR	95% CI
**Age**	0.311	0.996	0.987–1.005	-	-	-
**CDI therapy** (Reference = Vancomycin group)						
Fidaxomicin	0.825	1.029	0.800–1.323	-	-	-
**Inpatient treatment** (Reference = Outpatient treatment)	**< 0.001**	9.801	7.181–13.094	**< 0.001**	4.944	3.552–6.802
**Severe first CDI episode**	**< 0.001**	2.205	1.438–3.586	**0.038**	1.452	1.038–2.093
**Refractory CDI** (Reference = Recurrent CDI)	0.162	1.213	0.929–1.594	-	-	-
**Type of CDI acquisition** (Reference = Community-acquired CDI)						
Hospital-acquired CDI	**< 0.001**	3.197	2.292–4.392	**< 0.001**	1.802	1.333–2.412
Health system-acquired CDI	**0.018**	1.514	1.069–2.121	0.388	1.127	0.846–1.492
**Treatment on intensive care unit**	**< 0.001**	2.932	2.246–3.885	**< 0.001**	2.119	1.645 − 2.752
**Rehospitalisation**	**0.036**	1.405	1.033–1.946	**< 0.001**	1.576	1.256 − 1.992
**Subsequent CDI episode**	0.465	1.108	0.846–1.465	-	-	-
**Symptom reduction at day 10**	0.281	1.258	0.809–1.870	-	-	-

Note: *n* = 320; Akaike information criterium: 6,703; Bayes information criterium: 6,733; deviation: 218; likelihood quotient chi-square: 228.2 (*p* < 0.001)

Abbreviations: CI, confidence interval; GLM, generalised linear model; OR, Odds ratio; CDI, *Clostridioides difficile* infection

## Discussion

In this retrospective case-control study we analysed the clinical and health economic impact of fidaxomicin in immunocompromised patients with recurrent and refractory CDI treated in an in- and outpatient setting in Germany. With regard to the clinical outcomes, we found a significantly higher rate of symptom resolution at day 10 of CDI therapy as well as a significantly lower rate of CDI recurrences in the fidaxomicin group compared with the vancomycin group. While costs for CDI therapy were significantly higher in the fidaxomicin group, hospitalisation and overall direct treatment costs were comparable in both groups.

In the fidaxomicin registration trials, the clinical efficacy of fidaxomicin compared with that of vancomycin at the end of therapy did not differ significantly in the subgroup of patients with previous episodes of CDI [14]. Cornely et al. reported similar clinical cure rates in a general population [16], as did Rao et al. in hospitalised patients receiving concomitant antibiotics for concurrent infections [34]. In our study, we observed a significantly higher rate of symptom reduction on day 10 in patients treated with fidaxomicin than in those treated with vancomycin – indicating that fidaxomicin is more effective than vancomycin in patients with relevant pre-existing conditions, older age and current refractory or recurrent CDI. Corresponding to our results, a recent retrospective single-centre, real-world study investigating treatment failure based on a combined outcome defined as clinical failure, recurrent CDI within 30 days after the end of initial CDI treatment, or death attributable to CDI, reported that fidaxomicin was associated with a risk reduction of 63% in comparison with vancomycin [22]. Consistent with our findings, treatment with fidaxomicin was associated with significantly lower rates of CDI recurrence than vancomycin, as demonstrated in the studies by Louie et al. and Cornely et al. [14, 16], as well as in a subgroup analysis of Cornely et al. with patients with recurrent CDI [25]. A recent study demonstrated that fidaxomicin was significantly more effective in treating patients with CDI recurrence after the initial CDI episode and preventing subsequent CDI episodes in comparison with vancomycin, metronidazole or the combination of vancomycin and metronidazole [26]. Notably, our study stands out by demonstrating that these favourable outcomes were confirmed not only in patients with recurrent or refractory CDI, but also in patients aged over 65 years and in immunocompromised patients. We were further able to show that treatment with fidaxomicin significantly reduced the risk of a new CDI recurrence, whereas additional antibiotic treatment after CDI therapy had a significant effect on recurrence. Rao et al. demonstrated that patients receiving antibiotics simultaneously with CDI treatment had non-significantly higher rates of treatment response with fidaxomicin than vancomycin [34]. Although, fidaxomicin-treated patients demonstrated a higher rate of symptom reduction on day 10 and lower recurrence rates compared with vancomycin-treated patients, the overall hospital length of stay was similar between both groups. This is likely because more than half of the patients in both groups were admitted for reasons other than CDI, including management of complications and treatments related to the underlying comorbidities.

While the clinical findings of our total study cohort correspond to those of the subgroup of patients with recurrent CDI, the results could not be replicated in the subgroup of refractory CDI patients. This may be explained by the fact that this subgroup consisted of a smaller sample size as well as possible interactions of the premedication preceding the change in antibiotic treatment. In this context, it is also noteworthy that we observed a higher percentage of inpatient treatment as well as a longer median length of hospitalisation in the refractory CDI subgroup than in the recurrent CDI subgroup. While in our cohort, fidaxomicin and vancomycin were given for a median duration of 11 and 12 days, Rao et al. reported longer durations of 15 and 17 days in a cohort of patients with initial CDI treated with other non-CDI concomitant antibiotics [34]. Furthermore, as noted in the current ESCMID guideline, persistent diarrhoea may result from causes other than refractory CDI, which could not be reliably distinguished in this study. Therefore, the results of the refractory case subgroup should be interpreted with due regard to the limitations inherent in its definition [10].

Recurrent and refractory CDI not only represent a remarkable clinical burden but also entail a considerable economic burden on the healthcare system, which increases with each additional episode [35–38]. A recently published study suggested that health economic understanding of the use of fidaxomicin has not been fully achieved [27]. According to the ESCMID guideline, fidaxomicin treatment is recommended as 200 mg twice a day for ten days, resulting in costs of €1,700 per patient [10]. In our study population, the mean costs exceeded this value by €320. In the control group, mean costs for vancomycin and vancomycin tapered pulsed therapy were €976 (95% CI 838-1,130) and €1,272 (95% CI 730-2,063) per patient. The guideline recommends vancomycin 125 mg four times daily for ten days for the treatment of recurrent or refractory CDI which results in €384 [10]. When the costs of standard treatment were compared with those in our study population, mean costs in our study cohort were approximately €592 higher, thus reflecting real-world costs within the German healthcare system. The overall drug acquisition costs of fidaxomicin for recurrent or refractory CDI amounted to €2,412 and were significantly higher compared with the costs of €1,256 for vancomycin. This result was to be expected, as fidaxomicin is still under patent and drug acquisition costs are higher compared to metronidazole and vancomycin across Europe [39]. In contrast, the hospitalisation costs as well as overall direct treatment costs were comparable between the two groups. This is in line with similar durations of hospitalisation. Nevertheless, overall direct treatment costs were higher in the subgroup of patients with refractory CDI than in those with recurrent CDI. These findings may be due to the longer hospital stays in the refractory group. Our findings are consistent with those of a study assessing the cost-effectiveness and budget impact of fidaxomicin in comparison with vancomycin in patients with an increased risk of recurrence in Germany. The authors concluded that, even though the costs were higher, fidaxomicin was more effective than vancomycin because of lower rates of recurrence, especially in subgroups such as cancer patients, geriatric patients, and patients with previous CDI [35]. Similarly, Okumura et al. evaluated patients after treatment failure with metronidazole and showed that fidaxomicin, in comparison with vancomycin, was associated with higher acquisition costs, which were compensated for by reduced hospitalisation costs on the basis of lower recurrence rates and reduced costs of complications [40]. Similar results were found in further studies in Spain [41] and the United Kingdom [20].

Our multivariable GLM identified inpatient treatment, treatment in ICU, hospital-acquired CDI, rehospitalisation as well as a severe first episode of CDI as cost drivers affecting the overall direct treatment cost of recurrent/ refractory CDI. In comparison, a systematic review evaluating economic assessments of fidaxomicin, vancomycin and metronidazole identified cure rate, recurrence rate, the duration of hospitalisation, as well as drug and associated costs as essential cost drivers [42].

There are several limitations to our study, including its retrospective design, which is prone to errors due to missing data, even though patients with incomplete medical records were not included in this study. Furthermore, we cannot exclude the possibility that unmeasured comorbidities or temporal trends outside the scope of the study may have influenced the results – limitations that are well-recognised in retrospective case-control studies [43]. As the study period covered a period of 11 years, there were changes in the guidelines for the treatment of CDI during this time. By matching patients on the basis of the year of CDI diagnosis, we aimed to minimise potential bias related to temporal variations in treatment practices and disease management. Moreover, while groups were defined based on the first-line CDI therapy during the index episode of recurrent or refractory CDI, changes in therapy during follow-up were also considered to reflect real-world clinical practice. These therapy changes may have affected treatment effectiveness and the observed clinical and health economic outcomes. A few patients received FMT or bezlotoxumab in addition to fidaxomicin, but a sensitivity analysis indicated that their inclusion did not meaningfully affect the study outcomes. Furthermore, hospitalisation and drug acquisition costs were difficult to compare across studies due to differences in healthcare systems, reimbursement policies, and pricing structures between countries as well as varying methods for the calculation of the hospitalisation costs. Additionally, no data are available on the costs associated with CDI treatment for patients treated exclusively in an outpatient setting. Consequently, we could analyse only the number of outpatient visits as an indicator of healthcare resource utilization.

Our study has several strengths. First, our study has a multicentre, matched, real-world cohort design, which enabled robust and valid comparisons between the two groups. Second, we focused on a high-risk group of patients, including haematological, oncological, immunocompromised, and geriatric patients, which is still a poorly assessed group of patients, especially regarding recurrent and refractory CDI. Third, to the best of our knowledge, this study is the first to examine the health economic impact of fidaxomicin in high-risk patients with recurrent or refractory CDI and to identify major cost drivers.

## Conclusions

In conclusion, our study demonstrated that patients with advanced age and immunocompromised patients with recurrent and refractory CDI treated with fidaxomicin as first-line treatment had improved clinical outcomes compared with patients treated with vancomycin, with similar hospitalisation and overall treatment costs. Treatment costs of refractory CDI patients were higher than those of recurrent CDI patients. Future studies should focus on effective approaches to minimise identified cost drivers while optimizing treatment strategies for recurrent and refractory CDI, especially in at-risk patients.

## Supplementary Information

Below is the link to the electronic supplementary material.

Supplementary Material 1

## Data Availability

The authors are not permitted to disclose the study data beyond the research team. However, the data can be accessed through direct request to the corresponding author, and R scripts used to analyse the data can be made available on reasonable request.

## Abbreviations

CDI: *Clostridioides difficile* infection
CI: Confidence interval
FMT: Faecal microbiota transfer
G-DRG: German Diagnosis Related Groups
GLM: Generalised linear model
HR: Hazard ratio
ICU: Intensive care unit
InEK: Institut für das Entgeltsystem im Krankenhaus
IQR: Interquartile range
LOS: Length of stay
NAAT: Nucleic acid amplification test
OR: Odds ratio
rCDI: Recurrent or refractory *Clostridioides difficile* infection
