# Supplementary material for: Clinical and Pharmacoeconomic evaluation of Fidaxomicin in patients over 65 years of age and immunocompromised patients with recurrent and refractory Clostridioides difficile infection
Source: BMC Infect Dis. 2026 Jan 29;26:270. doi: 10.1186/s12879-026-12611-4 (PMC12874842; doi:10.1186/s12879-026-12611-4)
Supplement: Supplementary file 1 — Supplementary Material 1 [file 12879_2026_12611_MOESM1_ESM.pdf]

Supplementary Material - Clinical and pharmacoeconomic evaluation of fidaxomicin in patients over 65 years of age and immunocompromised patients with recurrent and refractory *Clostridioides difficile* infection

Recurrent *Clostridioides difficile* infection

Table S1 Clinical outcome of patients with recurrent *Clostridioides difficile* infection

	**Fidaxomicin group (n=110)**	**Vancomycin group (n=112)**	**p value**
**Days with CDI-associated diarrhea, median (IQR)**	4 (2-6)	4 (2-6)	0.203[a]
**Symptom reduction on day 10 of CDI therapy, n (%)**	108 (98)	95 (85)	**<0.001[b]**
**New CDI recurrence within 90 days, n (%)**	25 (23)	47 (42)	**0.002[b]**
**90-day mortality, n (%)**	13 (12)	13 (12)	0.899[b]
**Days of initial hospitalisation, median (IQR)**	20 (10-46)	26 (11-54)	0.361[a]

Abbreviations: IQR, interquartile range; CDI, *Clostridioides difficile* infection

[a] Mann-Whitney U Test; [b] Pearson's chi square test

Table S2 Direct treatment costs for patients with recurrent *Clostridioides difficile* infection in Euro

	**Fidaxomicin group (n=110)**	**Vancomycin group (n=112)**	**p value**
**Hospitalisation costs[l, b], n (%)**	79 (72)	80 (71)	0.949[d]
Costs per patient, mean (95% CI)	19,364 (13,490-25,239)	18,619 (13,191-24,049)	0.872[a]
**Hospitalisation costs (Year 2024)**			
Costs per patient, mean (95% CI)	19,539 (14,257-24,821)	20,231 (14,729-25,733)	0.844[a]
**Overall recurrent/ refractory CDI drug acquisition costs[c], n (%)**	110 (100)	110 (98)	0.498[e]
Costs per patient, mean (95% CI)	2,404 (2,228-2,591)	1,307 (1,085-1,543)	**<0.001**[a]
**Overall direct treatment costs[b, c], n (%)**	110 (100)	112 (100)	-
Costs per patient, mean (95% CI)	16,311 (11,749-20,873)	14,583 (10,443-18,723)	0.593[a]
**Overall direct treatment costs (Year 2024)**			
Costs per patient, mean (95% CI)	16,437 (12,254-20,619)	15,734 (11,493-19,976)	0.826[a]

Abbreviations: CI, Confidence interval; CDI, *Clostridioides difficile* infection

[l] n-values differ due to missing data; [a] Bootstrapped t-test (independent samples, two sided); [b] Based on G-DRGs from 2013 to 2023; [c] Based on Rote Liste® prices from 2024; [d] Pearson chi-square test (two-tailed); [e] Fisher´s exact test

Refractory *Clostridioides difficile* infection

Table S3 Clinical outcome of patients with refractory *Clostridioides difficile* infection

	**Fidaxomicin group (n=62)**	**Vancomycin group (n=61)**	**p value**
**Days with CDI-associated diarrhea, median (IQR)**	8 (5-10)	6 (4-12)	0.488[a]
**Symptom reduction on day 10 of CDI therapy, n (%)**	55 (89)	52 (85)	0.648[b]
**New CDI recurrence within 90 days, n (%)**	11 (18)	17 (28)	0.181[b]
**90-day mortality, n (%)**	12 (19)	9 (15)	0.497[b]
**Days of initial hospitalisation, median (IQR)**	40 (28-65)	37 (25-49)	0.191[a]

Abbreviations: IQR, interquartile range; CDI, *Clostridioides difficile* infection

[a] Mann-Whitney U Test; [b] Pearson's chi square test

Table S4 Direct treatment costs for patients with refractory *Clostridioides difficile* infection in Euro

	**Fidaxomicin group (n=62)**	**Vancomycin group (n=61)**	**p value**
**Hospitalisation costs[l, b], n (%)**	53 (85)	55 (90)	0.428[d]
Costs per patient, mean (95% CI)	19,694 (13,650-25,738)	19,487 (14,617-24,356)	0.960[a]
**Hospitalisation costs (Year 2024)**			
Costs per patient, mean (95% CI)	20,662 (15,334-25,990)	20,330 (16,219-24,440)	0.924[a]
**Overall recurrent/ refractory CDI drug acquisition costs[c], n (%)**	62 (100)	60 (98)	0.496[e]
Costs per patient, mean (95% CI)	2,412 (2,126-2,794)	1,164 (876-1,519)	**<0.001**[a]
**Overall direct treatment costs[b, c], n (%)**	62 (100)	61 (100)	-
Costs per patient, mean (95% CI)	19,247 (13,822-24,672)	18,715 (14,102-23,328)	0.870[a]
**Overall direct treatment costs (Year 2024)**			
Costs per patient, mean (95% CI)	20,074 (15,197-24,951)	19,475 (15,438-23,512)	0.841[a]

Abbreviations: CI, Confidence interval; CDI, *Clostridioides difficile* infection

[l] n-values differ due to missing data; [a] Bootstrapped t-test (independent samples, two sided); [b] Based on G-DRGs from 2013 to 2023; [c] Based on Rote Liste® prices from 2024; [d] Pearson chi-square test (two-tailed); [e] Fisher´s exact test

Patients without receiving FMT or bezlotoxumab as additional first-line therapy

Table S5 Sensitivity analysis excluding two patients who received FMT and five patients that received bezlotoxumab as first-line therapy for recurrent or refractory *Clostridioides difficile* infection, with two patients receiving both treatments in addition to fidaxomicin. After excluding these cases and their matched controls, the number of patients was 167 per group.

	**Fidaxomicin group (n=167)**	**Vancomycin group (n=167)**	**p value**
**Days with CDI-associated diarrhea, median (IQR)**	5 (3-8)	4 (3-9)	0.868[a]
**Symptom reduction on day 10 of CDI therapy, n (%)**	158 (95)	144 (86)	0.009[b]
**New CDI recurrence within 90 days, n (%)**	35 (21)	61 (37)	0.002[b]
**90-day mortality, n (%)**	25 (15)	21 (13)	0.481[b]

Abbreviations: IQR, interquartile range; CDI, *Clostridioides difficile* infection

[a] Mann-Whitney U Test; [b] Pearson's chi square test
